# Late p65 nuclear translocation in glioblastoma cells indicates non-canonical TLR4 signaling and activation of DNA repair genes

**DOI:** 10.1038/s41598-020-79356-1

**Published:** 2021-01-14

**Authors:** Isabele F. Moretti, Antonio M. Lerario, Marina Trombetta-Lima, Paula R. Sola, Roseli da Silva Soares, Sueli M. Oba-Shinjo, Suely K. N. Marie

**Affiliations:** 1 Laboratory of Molecular and Cellular Biology (LIM15), Department of Neurology, Faculdade de Medicina FMUSP, Universidade de Sao Paulo, São Paulo, SP Brazil; 2 Department of Internal Medicine, Division of Metabolism, Endocrinology and Diabetes, University of Michigan, Ann Arbor, MI USA

**Keywords:** Cancer, Cell biology, Immunology, Molecular biology

## Abstract

Glioblastoma (GBM) is the most aggressive brain primary malignancy. Toll-like receptor 4 (TLR4) has a dual role in cell fate, promoting cell survival or death depending on the context. Here, we analyzed *TLR4* expression in different grades of astrocytoma, and observed increased expression in tumors, mainly in GBM, compared to non-neoplastic brain tissue. TLR4 role was investigated in U87MG, a GBM mesenchymal subtype cell line, upon LPS stimulation. p65 nuclear translocation was observed in late phase, suggesting TLR4-non-canonical pathway activation. In fact, components of ripoptosome and inflammasome cascades were upregulated and they were significantly correlated in GBMs of the TCGA-RNASeq dataset. Moreover, an increased apoptotic rate was observed when the GBM-derived U87MG cells were co-treated with LPS and Temozolomide (TMZ) in comparison to TMZ alone. Increased TLR4 immunostaining was detected in nuclei of U87MG cells 12 h after LPS treatment, concomitant to activation of DNA repair genes. Time-dependent increased *RAD51*, *FEN1* and *UNG* expression levels were confirmed after LPS stimulation, which may contribute to tumor cell fitness. Moreover, the combined treatment with the RAD51 inhibitor, Amuvatinib in combination with, TMZ after LPS stimulation reduced tumor cell viability more than with each treatment alone. In conclusion, our results suggest that stimulation of TLR4 combined with pharmacological inhibition of the DNA repair pathway may be an alternative treatment for GBM patients.

## Introduction

Toll-like receptor 4 (TLR4) is part of the receptor family for innate immunity that first recognizes endogenous (damage-associated molecular patterns–DAMPs) and exogenous (pathogen-associated molecular patterns-PAMPs) molecules^1^. This family is composed of 10 known receptors in humans, which are structurally similar (TLR1-10)^2^. Depending on the context, TLR4 activation may induce a pro-inflammatory and pro-survival response, which translates into a proliferative phenotype or may induce an anti-inflammatory response leading to cell death^2,3^.

TLR4 downstream signaling includes two distinct pathways: the myeloid differentiation primary response gene 88 (MyD88; "canonical" pathway) and TIR-domain-containing adapter-inducing interferon-β (TRIF; "non-canonical" pathway). Signaling through the canonical TLR4 leads to the activation of the transcription factor nuclear kappa B (NF-κB) by means of the translocation of its heterodimeric complex p50/p65 to the nucleus^4^. Once in the nucleus, NF-κB induces the transcription of pro-inflammatory genes coding for interleukins 6 (IL6) and 1β (IL-1β), tumor necrosis factor (TNF), adhesion molecules, and chemokines^5–7^, as well as genes coding for a proliferative response^8–12^. On the other hand, the non-canonical pathway consists of the TLR4 internalization to the endosome compartment by a phosphoinositide 3-kinase (PI3K)-dependent mechanism^13^. This process results in the activation of TRIF, which promotes an anti-inflammatory response by inducing the expression of interferon type I, interferon regulatory factor 3 and 7 (IRF3/7), and IL-10^2,14^. TRIF may also trigger a cell death pathway by interacting with receptor interacting protein kinase 1 and 3 (RIPK1, RIPK3), and the fas adaptor death domain (FADD), which in turn activate caspase 8 (CASP8), leading to apoptosis. In the absence of CASP8, the necroptosis pathway is activated^15,16^.

TLR4 stimulation may also lead to NACHT, LRR, and PYD domains-containing protein 3 (NLRP3) inflammasome activation by either MyD88 or the TRIF-dependent pathway^17,18^. The NLRP3 inflammasome complex comprises NLRP3, a sensing molecule with a pyrin domain (PYD), which interacts with an adaptor protein (ASC) with a CARD domain. The resulting combined ASC-CARD domain interacts with pro-CASP1^19^, which cleaves and releases interleukin-1 family members such as IL-1β and interleukin-18 (IL18)^20^.

Inflammatory cells in a solid tumor microenvironment may exhibit a pro- or anti-inflammatory profile depending on the stimuli^21^. Moreover, inflammatory receptors such as TLR4 are also expressed in tumor cells, adding another layer of complexity that results from potentially distinct effects of TLR4 downstream signaling in each cell compartment^22^. We previously demonstrated an increased TLR4 expression in human astrocytoma, particularly glioblastoma (WHO-grade IV astrocytoma). Glioblastoma (GBM) is the most common and the most aggressive primary brain malignancy in adults^23^, and due to its relevant heterogeneity and the invasive feature, the outcomes of current therapeutic strategies have remained almost invariably lethal. In fact, the standard of care, including surgical tumor cytoreduction followed by radiotherapy and adjunct chemotherapy with Temozolomide (TMZ), and several combinatorial rescue trials have not improved the median overall survival time of approximately 15 months^24^. Among the GBM molecular subtypes, the mesenchymal (MES) subtype, which harbors *neurofibromin 1 (NF1)* and *RB transcription corepressor 1* (*RB1*) mutations, presents the poorest outcome, compared to the proneural (PN) subtype harboring somatic mutations in *tumor protein p53 (TP53), platelet-derived growth factor receptor A (PDGFRA),* and *isocitrate dehydrogenase 1 (IDH1),* and to the classical subtype with *epidermal growth factor receptor (EGFR)* mutations^25,26^.

In this context, we aimed to analyze the impact of TLR4 stimulation in a MES-GBM tumor cell. We worked with the hypothesis that activating the TLR4 downstream cascade might activate a cell death pathway and contribute to a better outcome for GBM patients, mainly with the MES subtype.

## Results

### TLR4 expression in human astrocytoma

**Figure 1:**
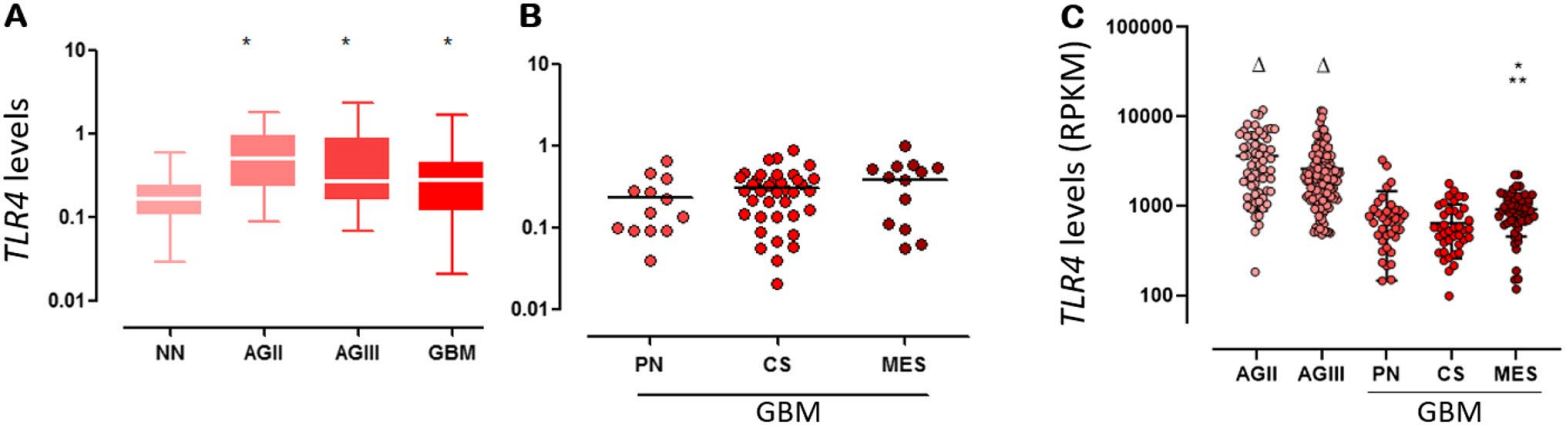
The *TLR4* mRNA expression level was upregulated in human astrocytoma. (**A**) Box plot representation of *TLR4* expression levels in our cohort of different astrocytoma malignant grades (AGII, AGIII, and GBM) and non-neoplastic (NN) brain samples (*p < 0.05 Kruskal–Wallis and Dunn tests). *TLR4* expression levels (2^−ΔCt^) are log10 transformed, and the horizontal bars represent the median values. (**B**) The expression levels of *TLR4* in the GBM molecular subtypes: proneural (PN), classical (CS), and mesenchymal (MES) in our cohort. (**C**) *TLR4* expression analysis in the TCGA RNASeq data set is demonstrated by reads per kilobase per million mapped reads (RPKM), transformed in log10, including values for AGII, AGIII and GBM molecular subtypes. AGII and AGIII presented higher expression values (Δ) compared to GBM, and MES subtype presented significant higher than CS (*) and PN (**)(*p* = 0.001, Kruskal–Wallis, *p* = 0.001 Dunn tests).

The upregulation of plasmatic membrane TLRs have been previously demonstrated in astrocytoma, particularly in GBM by our group^27^. Here, we first recapitulated *TLR4* expression in our cohort of 140 human astrocytoma of different grades of malignancy (26 AGII, 18 AGIII, and 96 GBM compared to 22 non-neoplastic [NN] brain tissue), and we next analyzed TLR4 signaling pathways. *TLR4* expression was significantly higher in AGII, AGIII, and GBM when compared to NN (*p* < 0.05, Kruskal–Wallis and Dunn tests). Interestingly, among GBM molecular subtypes (14 PN, 36 CS, and 16 MES)^26^, *TLR4* expression was higher in MES than in PN and CS subtypes, however a statistical significance was not reached in our cohort due to the small number of cases in each subtype. Then, we validated this result in a larger dataset of the TCGA cohort, and a significant difference of *TLR4* expression was confirmed among GBM subtypes (*p* < 0.0001 Kruskal–Wallis test), being higher in MES subtype compared to PN and to CS subtypes (*p* = 0.001 for both comparisons, Dunn test) (Fig. 1) Lower grade astrocytomas (AGII and AGIII) presented *TLR4* higher expression levels when compared to GBM samples (*p* = 0.001 for both comparisons, Dunn test).

### TLR4 canonical signaling pathway in U87MG cells

**Figure 2:**
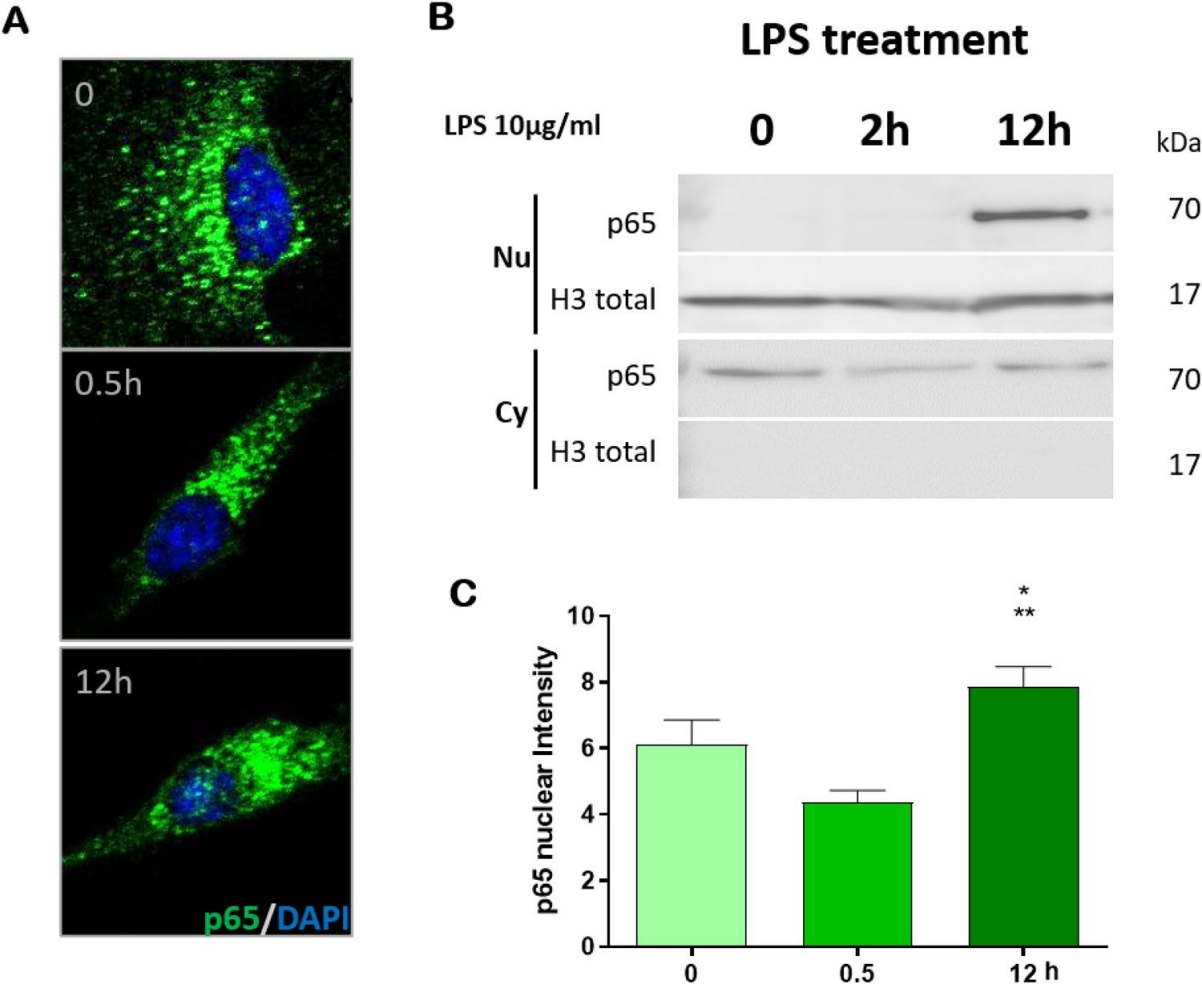
Translocation of the p65 subunit of NF-kB to the nucleus of U87MG cells after LPS treatment. (**A**) Immunofluorescence confocal images at three different time points (0, 0.5, 12 h) of U87MG cells treated with LPS showed p65 translocation to nucleus after 12 h of treatment. Cells were incubated with anti-p65, followed by a secondary antibody anti-rabbit-Alexa Fluor 488, in green, and with DAPI, in blue. 400 × magnification (**B**) Western blot analysis of p65 with nuclear (Nu) and cytoplasmic (Cy) extracts of U87MG cells after 2 and 12 h of LPS treatment. Histone H3 was used as a loading control for nuclear extract. (**C**) Quantification of nuclear fluorescence intensity of p65 staining (n = 30) at the three time points (*p < 0.05 compared to 0 h and **p < 0.05 compared to 0.5 h by one-way Anova, Tukey test).

To access TLR4 role in a GBM cell line, we treated U87MG cells with LPS and observed NF-κB activation aiming to analyze the canonical TLR4 signaling pathway. The NF-κB translocation to the nucleus was assessed by the presence of the p65 subunit in the nucleus. In immune cells, NF-kB translocation to the nucleus has been detected 30–100 min after TNF stimulation^28^. However, in our experiment the nuclear translocation was not observed at 30 min or 2 h after LPS stimulation, the time interval expected for NF-κB translocation to occur through the MyD88/TRAF6 canonical pathway. It occurred only after 12 h, being considered a late translocation, as demonstrated by immunofluorescence analysis and Western blotting (Fig. 2A–C).

### TLR4 non-canonical signaling pathway in U87MG cells

**Figure 3:**
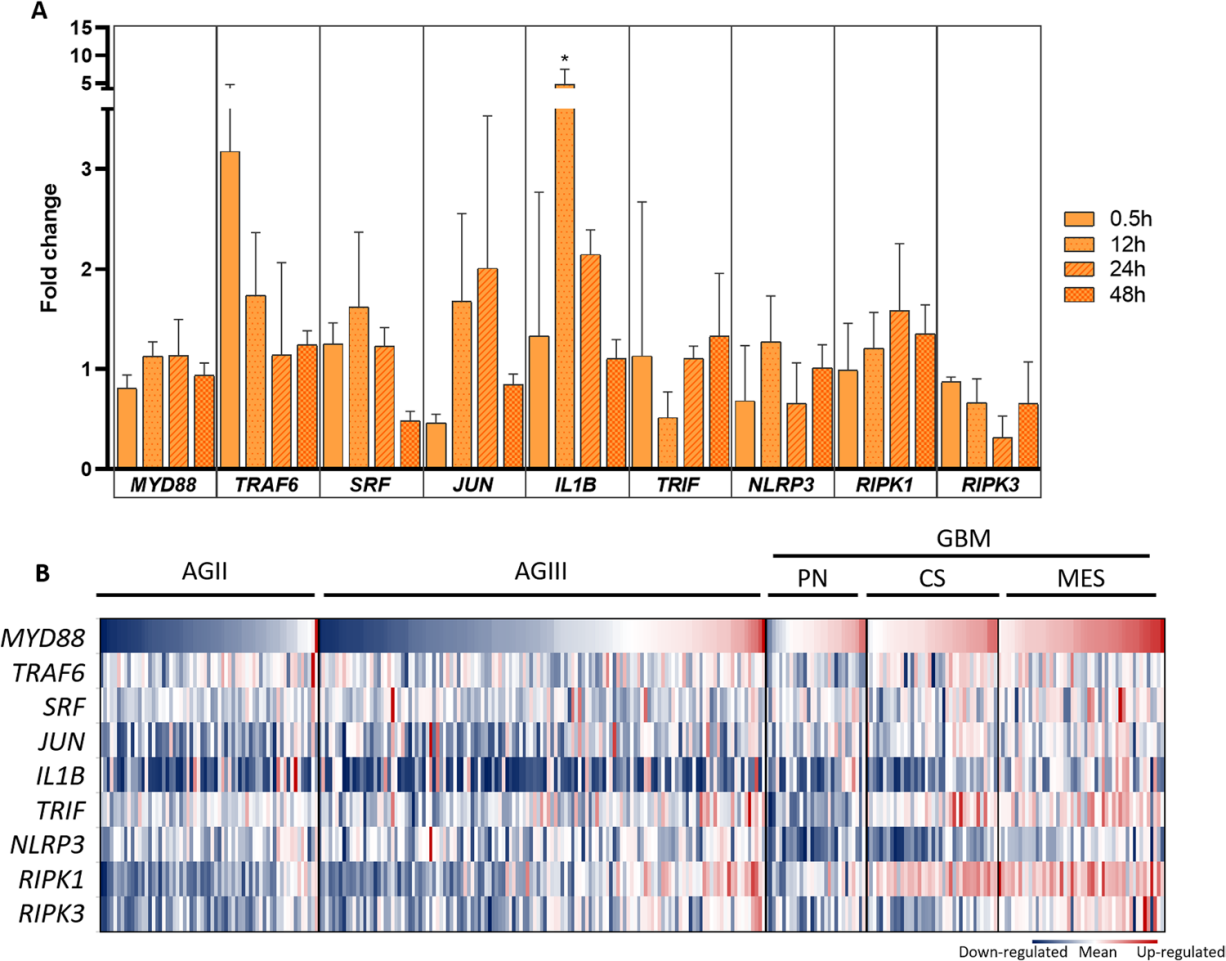
Canonical and non-canonical gene expression profile after LPS stimulation of U87MG cells and of TCGA astrocytoma RNASeq data. (**A**) *MYD88, TRAF6, JUN, SRF, IL1B, TRIF, NLRP3, RIPK1, RIPK3* expression ratio with the non-treated cell were accessed by qRT-PCR, at different time points (0.5, 12, 24, 48 h) in three independent experiments. The fold change values were calculated by the ratio of the value obtained by 2^−ΔCt^ formula of treated cells compared to control cells (time point 0). (**B**) Heatmap of the RPKM values from the TCGA RNASeq dataset, normalized by z-score for the selected genes of astrocytoma cases of different malignant grades (AGII, AGIII, and GBM). GBM cases were subdivided by molecular subtypes proneural (PN), classical (CS), and mesenchymal (MES). Upregulated values are in red and downregulated in blue.

To further investigate the TLR4-signaling pathways after LPS stimulation of U87MG cells, considering the late translocation of NF-κB to the nucleus, we performed a qRT-PCR for the *MYD88*, and *TRAF6* of the canonical pathway*; SRF*^9^, and *JUN*^29^ for the proliferative response; *TRIF*^30^ *for the endosome; NLRP3*^17^ and *IL1B*^31^ for inflammasome; and *RIPK1* and *RIPK3* for ripoptosome pathways^32^, at different time points: 0, 0.5, 12, 24 and 48 h, in three independent experiments. *MYD88*, *SRF*, *JUN*, *IL1B*, *NLRP3, and RIPK1* expression levels increased after 12 h of LPS stimulation, and *IL1B* expression reached the largest fold change of 5.83 times compared to basal expression level. Interestingly, *TRAF6* was the only analyzed target presenting a peak of increased expression within 30 min of stimulation (*p* = 0.052, One-way Anova post-hoc Tukey test), but it was not related to the NF-kB translocation to the nucleus, as shown by the Western blot analysis (Fig. 2B). The mRNAs expression alterations at 12 h after LPS stimulation corroborated the activation of TLR4-inflammasome, and -ripoptosome pathways, with increase of *SRF*, *JUN* and particularly *IL1B* (*p* < 0.05 One-way Anova, *p* = 0.012 Tukey test) transcripts levels. (Fig. 3A). We next checked the expression level of these targets in the TCGA RNASeq dataset to validate the activation of these pathways in human astrocytoma (Fig. 3B). In fact, significant upregulations of *MYD88*, *SRF*, *JUN*, *IL1B, TRIF*, *RIPK1*, *NLRP3* were detected in GBM cases compared to lower grade astrocytomas (AGII and AGIII) (Supplementary Fig. 1). When the expression pattern of these genes was compared among the GBM subtypes, MES subtype presented higher *MYD88*, *IL1B*, *RIPK3* and *NLRP3* expression levels than PN and CS subtypes (Supplementary Fig. 1), in a similar pattern to *TLR4* expression (Fig. 1C).

Therefore, these observations of the TCGA dataset were convergent to U87MG expression profile after LPS stimulation, indicating upregulation of inflammasome and ripoptosome pathways in GBM, particularly in MES subtype.

**Figure 4:**
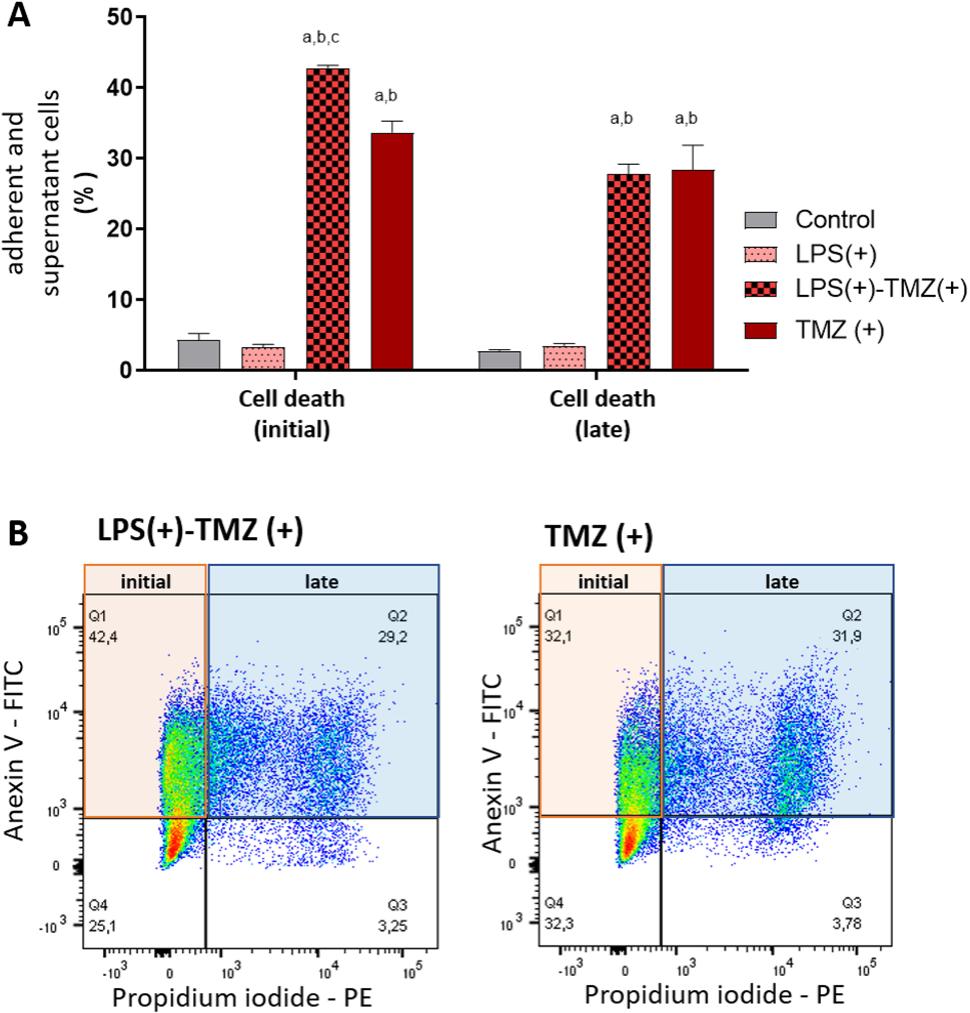
Cell death functional assay by flow cytometry. (**A**) U87MG cells were treated for 48 h with LPS, TMZ, and LPS together with TMZ. The cells were incubated with annexin V and propidium iodide (PI) and evaluated by flow cytometry for the adherent and supernatant cells. Statistical analysis was performed using a Two-way Anova *p* < 0.001 followed by Bonferroni post-test, where significance was ^a^*p* < 0.001 compared to non-treated cells (control group), ^b^*p* < 0.001 compared to cells treated with LPS, and ^c^*p* < 0.001 in comparison to cells treated with TMZ. (**B**) Representative scatter plots of Annexin V-FITC/PI staining of U87MG cells with TMZ alone or in combination with LPS. The initial cell death phenotype was indicated when the cells were positive by annexin V (orange). Late cell death was given by the positivity for annexin V and PI (blue).

As a next step, we checked whether the activation of TLR4 by LPS presented a co-stimulatory effect to TMZ, the alkylating agent used in the standard of care of GBM patients. Interestingly, U87MG cells presented a more significant early cell death after combined stimulation than either TMZ- or LPS-alone treatment after 48 h (Fig. 4A). We observed an approximately 10% increase in the initial cell death by the combined treatment in comparison to the TMZ single treatment (Fig. 4B). No differences among the stimulation conditions were observed for the number of cells in late cell death.

### TLR4 localization in nuclei of U87MG cells

**Figure 5:**
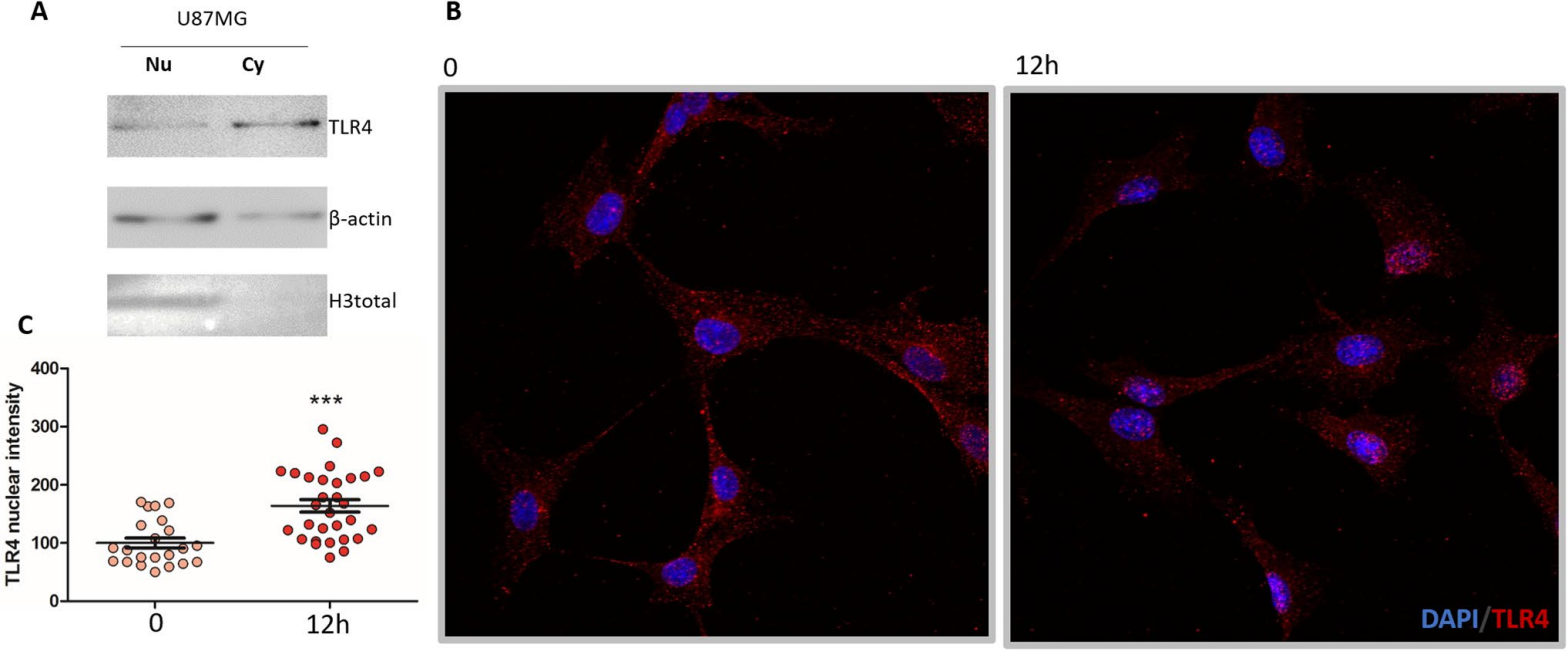
TLR4 localization in U87MG cells. (**A**) TLR4 presence was observed in nuclear (Nu) and cytoplasmic (Cy) protein fractions of U87MG cell line by Western blot analysis. Total H3 protein was used for nuclear protein enrichment control and β-actin for protein loading control. (**B**) The presence of TLR4 was detected in cytoplasmic and nuclear compartments in U87MG cells by immunofluorescence. TLR4 staining in the nucleus increased after 12 h of LPS stimulation. Cells incubated with anti-TLR4-secondary antibody anti-mouse-Alexa Fluor 568, in red, and with DAPI, in blue. 200 × magnification. (**C**) Values for nuclear fluorescence intensity for each analyzed cell n = 23 at 0 h and n = 25 at 12 h (***p < 0.001 compared to 0 h, Mann-–Whitney).

We also analyzed TLR4 protein distribution within U87MG cells upon 12 h of LPS stimulation compared to control. Unexpectedly, immunofluorescence analysis showed a granular pattern of TLR4 expression in nuclei in addition to its distribution in cytosol and plasma membrane in these cells (Fig. 5B), with increased immunostaining after 12 h of LPS stimulation (Fig. 5C) (*p* < *0.05,* Mann Whitney). Such nuclear TLR4 distribution was also confirmed by Western blot in non-treated cells (Fig. 5A).

### Increased mRNA expression of DNA repair genes related to TLR4 stimulation

**Figure 6:**
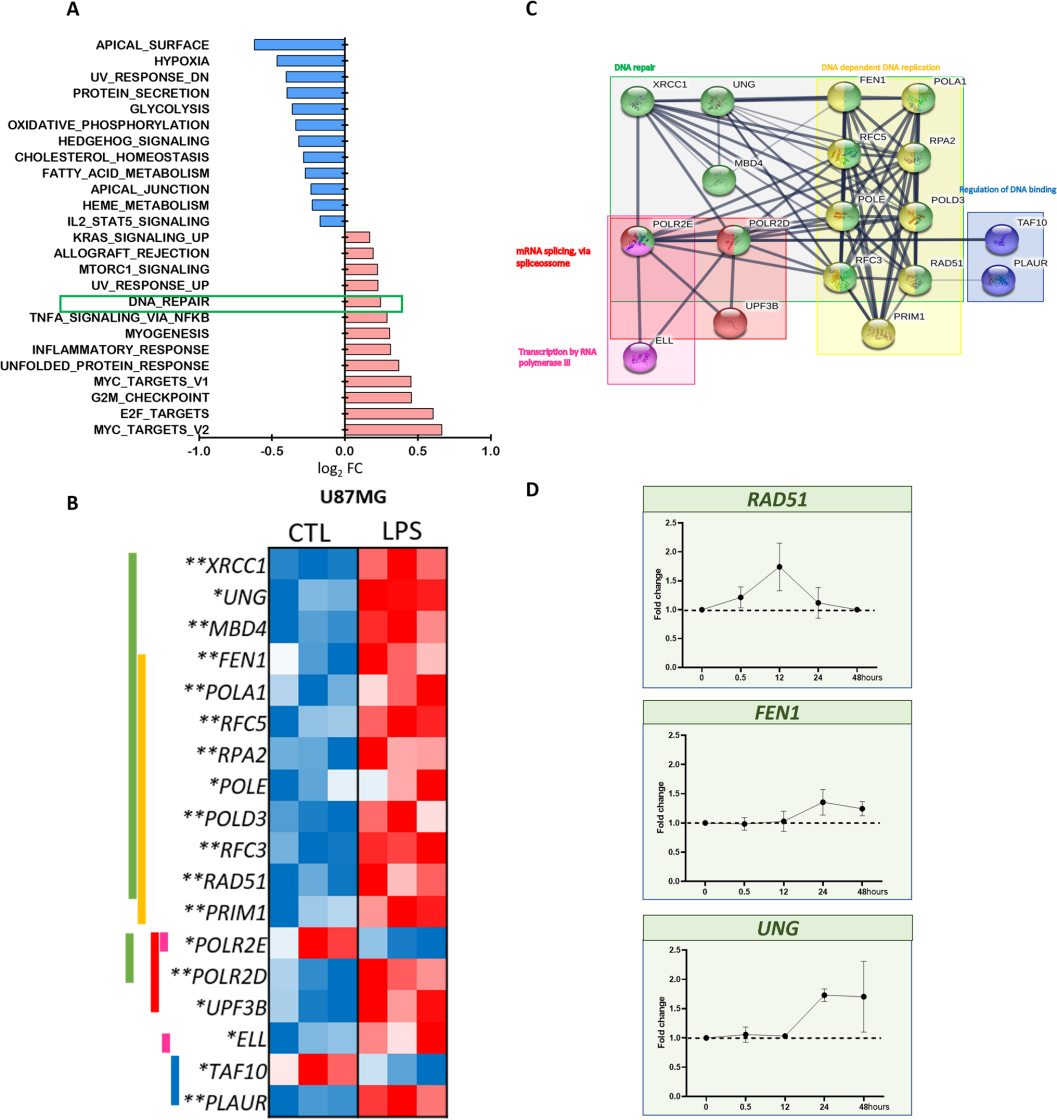
Transcriptome analysis of U87MG cell line treated with LPS showed upregulation of genes related to DNA repair when compared to non-treated condition (CTL). (**A**) 25 gene sets from “Hallmark” gene sets from the MSigDB database were significantly enriched upon LPS treatment *(*adjusted *p* < 0.1*)*, bars represent the value for the log_2_FC, and the DNA repair gene set is highlighted in the red box*.* (**B**) Heatmap representing the expression of 18 genes coding for proteins with biological functions for DNA repair. Each group was analyzed in triplicates for CTL and LPS treated U87MG cells. *p value < 0.05 ** adjusted *p* < 0.1. (**C**) The differentially expressed genes were plotted in a network according to the biological function (Gene Ontology Resource) (accessed: 2020–02-10 10.5281/zenodo.2529950) by the String Consortium tool. (**D**) *RAD51, UNG, FEN1* levels in UM87MG cell stimulated with LPS at different time points. The fold change values were calculated by the ratio with the control cells (time point 0) for the 2^−ΔCt^ formula. The dotted line represents the ratio value for the control (FC = 1).

To better understand the role of TLR4 in the nucleus, we performed RNASeq in U87MG cell lines after 12 h of LPS stimulation and compared to non-treated cells. This transcriptomic analysis showed 286 upregulated genes and 232 downregulated genes (logFC >|0.5|, *p* < 0.05, and adjusted *p* < 0.1). The enrichment analysis by Hallmark gene sets^33^ showed statistical significance for 25 pathways, including the pathway for DNA repair (HALLMARK_DNA_REPAIR), that was upregulated in the treated cells with a logFC of 0.244 (Fig. 6A). Eighteen differentially expressed genes included in this Hallmark group were detected, 16 genes upregulated and 2 genes downregulated after LPS treatment compared to control cells (Fig. 6B). These genes were grouped according to their biological function by GO classification as: DNA repair, DNA dependent DNA replication, regulation of DNA binding, mRNA splicing via spliceosome and transcription by RNA polymerase III. The analysis with the String Consortium tool showed high connectivity among them (Fig. 6C). Additionally, in the TCGA dataset of astrocytoma, their expressions were significantly higher in GBM compared to lower grade astrocytoma (AGII and AGII), (*p* < 0.0001, Kruskal–Wallis test), except for *XRCC1* and *POLA1.* Among them, *MBD4* (methyl-CpG binding domain 4), *PLAUR* (urokinase plasminogen activator receptor), *POLE* (DNA polymerase epsilon, catalytic subunit) and *RAD51* (RAD51 recombinase) expressions were significantly higher in GBM-MES subtype when compared to CS and PN subtypes (*p* < 0.05 Dunn test for all comparisons) (Supplementary Fig. 1). The DNA repair gene expressions correlated positively among themselves, particularly *RAD51* with *FEN1* (flap structure-specific endonuclease 1) (*r* = 0.632*, p* = 0.0001) and with *UNG* (uracil DNA glycosylase) *(r* = 0.263*, p* = 0.003) in GBM. In MES subtype, a stronger correlation was observed between *RAD51* and *FEN1* expression (r = 0.758, *p* = 0.0001, Spearman test).

Considering these correlation findings in the TCGA dataset and our observations of differential expression after LPS stimulus in U87MG cells, we checked the expression level of *RAD51*, *FEN1*, and *UNG* of U87MG cells after 0.5, 12, 24 and 48 h after LPS stimulation. These three genes presented an increase of their expressions in 24 h, with further increase of *UNG* expression in 48 h (Fig. 6D). Thus, these findings corroborated our transcriptomic data of DNA repair related genes upregulation upon LPS stimulation.

### Decreased viability after RAD51 inhibition and LPS stimulation of U87MG cells

**Figure 7:**
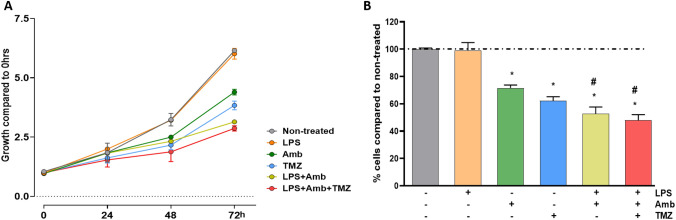
U87MG cell viability with LPS, Amb and TMZ combined treatment. (**A**) Cell viability curves with values normalized by the 0 h values at 0, 24, 48, 72 h with different treatments: LPS-alone, Amb-alone, TMZ-alone, LPS + Amb, LPS + Amb + TMZ compared to non-treated cells. (**B**) The percentage of cell viability decrease compared to non-treated cells after 72 h treatment. The bar graphs represent the fluorescence of viable cells normalized by the mean of non-treated cells (wherein *p ≤ 0.001 compared with non-treated and #*p* ≤ 0.001 compared with Amb-alone and TMZ-alone, Two-way Anova, followed byTukey post-hoc test).

Considering the upregulation of *RAD51* expression after TLR4 stimulation with LPS, we chose Amuvatinib (Amb), a RAD51 inhibitor tested in clinical trial for advanced solid tumors^34^ to check the impact on U87MG cells (Fig. 7A).

LPS treatment alone did not alter U87MG cell viability, however it significantly decreased with Amb single treatment (compared to non-treated in 48 and 72 h, *p* < 0.001). TMZ single treatment led to a sharper viability decrease in 72 h (compared to non-treated in 48 and 72 h, *p* < 0.001; compared to Amb in 72 h, *p* < 0.001). Moreover, a more pronounced decrease of cell viability was observed when Amb was combined to LPS stimulation (compared to non-treated in 48 and 72 h, *p* < 0.001; compared to Amb in 72 h, *p* < 0.001; compared to TMZ in 72 h, *p* < 0.001), which was comparable to Amb and TMZ combined treatment after LPS stimulation (compared to non-treated in 48 and 72 h, *p* < 0.001; compared to Amb in 72 h, *p* < 0.001; compared to TMZ in 72 h, *p* < 0.001) (Fig. 7B).

## Discussion

In view of TLR4 dual role in cellular fate, which is a direct consequence of both the stimuli and microenvironmental context, we aimed to evaluate TLR4 molecular mechanism in human GBM cells. First, we determined *TLR4* expression in 140 human astrocytoma samples of different malignant grades. We observed that *TLR4* expression was higher in astrocytoma samples when compared to non-neoplastic samples. Moreover, when we focused on GBM samples, the analysis of the TGCA RNASeq dataset revealed that *TLR4* expression was higher in the MES subtype, with the poorest outcome when compared to PN and CS subtypes.

The function of TLR4 has been extensively described in immune cells^35^. Nonetheless, TLR4 role in the tumor cell compartment is still lacking. To analyze TLR4 signaling pathways in GBM cells, the U87MG cell line, harboring *NF1* mutation^36^, was chosen as a model for the MES-GBM subtype^25^.

We observed that stimulation of U87MG cells with LPS, a traditional TLR4 agonist that activates pro-inflammatory pathways^37^, led to NF-kB (p65) nuclear translocation after 12 h. This was considered a late response because both tumor necrosis factor-alpha (TNFα) and LPS stimulation have been described to induce NF-κB translocation to the nucleus through the MyD88/TRAF6 canonical pathway within 30 min to 2 h^38^. Additionally, hyaluronic acid, a component of the brain extracellular matrix, has been described to trigger TLR4-NF-κB pathway in GBM stem-like cell differentiation and maintenance in a proliferative astrocytic precursor state^22^. Furthermore, angiogenin has also induced a proliferative phenotype in U87MG cells through NF-κB translocation to the nucleus^39^. In opposition to these previous observations, an unequivocal proliferative effect was not observed in our experiments with U87MG. In another report, TLR4 was able to suppress tumor growth by decreasing the expression of a transcription factor responsible for the maintenance of cancer stem cells, the retinoblastoma binding protein 5^40^.

A late NF-κB translocation to nucleus was observed in U87MG cells stimulated by LPS, and the gene expression profile of our experiments was compatible to activation of inflammasome and ripoptosome pathways. TRIF was described to interact with TRAF6, leading to a late-phase NF-κB activation. Moreover, TLR4 signaling activates the TRIF-RIPK3 complex^41^ and leads to apoptosis in the presence of CASP8, or to necroptosis in its absence^32^. However, CASP8 activation can be prevented by its inhibitor, c-FLIP, or by blocking TLR4-pathway and inhibiting apoptosis^42^. Following TLR4 internalization, TRIF mediates the activation of inflammasome complexes^43^. Two distinct inflammasome pathways have been described: a pro-proliferative through NLRP3 overexpression^44^ and a cell death signaling pathway^45^, a pyroptotic type of cell death triggered by persistent stimulation of IL-1β^46^. Additionally, the TLR4 pathway has been described to trigger apoptosis in glioma in a TRIF-dependent pathway^47^. Our transcriptomic data of U87MG cells 12 h after LPS stimulation and the TCGA RNASeq dataset of astrocytoma showed upregulation of genes related to inflammasome and ripoptosome pathways, namely *MYD88*, *NLRP3*, *RIPK1* and *RIPK3*, and also *SRF*, *JUN* and *IL1B*, the downstream regulated genes.

In this context, an increase of apoptosis of LPS stimulated U87MG cells was expected, however only an increase of approximately 10% in early apoptosis 48 h after the combined treatment of TMZ and LPS was observed. Moreover, we detected an increase of TLR4 distribution in the nucleus of U87MG, 12 h after LPS stimulation. In fact, portions of the TLR4 protein were predicted by computational analysis to be in the nucleus^48^, and such nuclear distribution has already been reported in pancreatic cancer cells^49^, as well as in non-neoplastic rat cells^50^. Additionally, the combined TLR4 expression in nuclei and in cytosol has been associated with poor prognosis and metastasis^51^.

In an attempt to understand the mechanisms that lead to the low rate of apoptosis, and the role of TLR4 in the nucleus, NGS transcriptomic analysis of U87MG cells after 12 h of LPS stimulation was performed. Interestingly, among the enriched gene sets differentially expressed after TLR4 activation we found the DNA repair-related genes. Particularly, *UNG, FEN1* and *RAD51* presented a time-dependent pattern of upregulation after LPS stimulation in U87MG cells. Considering that the activation of DNA repair cascade may increase tumoral cell fitness, with consequent enhancement of tumor cell survival, we tested the impact of inhibiting this cascade in this model of TLR4-LPS stimulation. In fact, such strategy has already been proposed as targeting the phosphorylation of H2AX, a histone acting for the assembly of repair foci, through modification of TLR4 pathway^22^. Moreover, the activation of DNA repair signals has been associated with TMZ resistance, including the MGMT (O-6-methylguanine-DNA methyltransferase) repair system, which catalyzes the transference of the methyl group to the cysteine residue of the MGMT protein^24^. Therefore, targeting DNA repair proteins has been pursued as interesting cancer therapeutic strategy.

Among the DNA repair related genes identified in our transcriptomic data, we selected RAD51 as its inhibition has already shown antitumor activity when combined with other therapeutic agents^34^. RAD51 has a role in the DNA repair pathway of homologous recombination (HR), and it forms a complex in a single strand DNA break, allowing the homology search and the HR process to continue^52^. Amuvatinib (Amb) is a multi-kinase inhibitor, including RAD51, and it has been tested in a clinical trial for advanced solid tumors^34^. Additionally, in several GBM cell lines, Amb had a radiosensitization effect and delayed tumor growth, as in U87MG subcutaneous xenograft mice model^53^. Convergently to this previous finding, we observed a significant reduction of U87MG cell viability with Amb treatment after TLR4-LPS stimulation, in a similar fashion as the combined Amb and TMZ treatment after TLR4-LPS stimulation. Furthermore, TLR4 protective role against tumorigenesis by downregulation of DNA damage repair proteins was also described in a mouse hepatocellular carcinoma^54^. Additionally, the absence of TLR4 was associated with DNA damage resistance after UV irradiation in mice skin cells^55^. These cumulative pieces of evidence suggest TLR4 as a DNA repair pathway regulator due to its capacity to recognize DAMPs and to generate an auto-paracrine signal in the presence of DNA damage^56^. Therefore, we speculate that the reduction of tumor cell viability by TLR4 stimulation combined with the downregulation of DNA repair genes may be an attractive complementary cancer therapeutic strategy, and a treatment alternative for tumor TMZ resistance, as hypermutated TMZ status^57^. Further clarification of the involved mechanisms might enable TLR4-targeted therapies to be combined with the current standard of care to improve GBM patient outcomes.

## Materials and methods

### *TLR4* expression in human astrocytoma and GBM cell line

Biological samples were collected during the neurosurgical procedure by the Neurosurgery Division of the Neurology Department of the Hospital das Clinicas, Faculdade de Medicina, Universidade de Sao Paulo (FMUSP) after informed and written consent from the patients following the Institutional Ethical Committee guidelines (process number: 691/05). The present study was approved by the HCFMUSP (process number: 059/15) and the FMUSP, ethical committee (process number: 278/15). The samples were snap frozen in liquid nitrogen and necrotic, gliotic, and non-neoplastic areas were previously macrodissected to guarantee presence of more than 90% of tumor cells in the processed tumor fragments as described previously^57^.

Biological samples and cell line total RNA were extracted using the RNeasy Mini Kit (Qiagen, Hilden, Germany) following the manufacturer instructions. Purity and concentration were analyzed by NanoDrop (Thermo Fisher Scientific, Carlsbad, CA, USA), and 1.8–2.0 values for the absorbance ratio 260/280 were considered satisfactory. Electrophoresis in agarose gel was done to check RNA quality.

For the reverse transcription, 1 µg of total RNA of each sample was used. Treatment with DNase I (FPLC-puro, GE Healthcare, Uppsala, Sweden) was performed, and RNA was reversely transcribed with random primers, oligodT oligonucleotides RNase inhibitor, and SuperScript III (Thermo Fisher Scientific). Lastly, the cDNA was treated with RNase H (GE Healthcare) and diluted in TE (Tris/EDTA) buffer.

We analyzed 22 non-neoplastic (NN) brain samples obtained from temporal lobectomy of epilepsy surgery, 26 astrocytoma grade II (AGII) samples, 18 astrocytoma grade III (AGIII) samples, and 96 GBM, classified 14 as PN, 38 CS and 16 MES subtype.

### Cell culture

U87MG human GBM cell lineage was acquired from ATCC and authenticated by short tandem repeats (STR) analysis using the GenePrint 10 System (Promega, Madison, WI, USA). Cells were cultured in monolayer with DMEM medium (Dulbecco's Modified Eagle's Medium) (Thermo Fisher Scientific) supplemented with 10% fetal bovine serum and 100 µg/ml streptomycin and 100 IU/ml penicillin. The cells were kept in an incubator at 37 °C with 5% CO_2_. U87MG cell line presents Neurofibromin 1 (NF1) mutation, with molecular pattern of MES subtype^36^.

### Western blotting

To analyze NF-kB activation by TLR4 stimulation, the translocation of the subunit p65 to the nucleus was evaluated by Western blot. Cytoplasmic and nuclear protein extractions were performed for U87MG cells after LPS treatment for 2 and 12 h. The cells were lysed with a buffer solution containing light detergents to separate the cytoplasmic proteins: Igepal-CA-630 (0,5%), Triton X-100 (0,25%), glycerol (10%) (Thermo Fisher Scientific), 1 mM EDTA, 50 mM Hepes, and 140 mM NaCl. After centrifugation, nuclei were lysed with a buffer containing strong detergent and sonicated 50 mM Tris–HCl, 10 mM EDTA and 1% SDS. The Western blotting was performed with the loading of 40 µg proteins in a 4–12% polyacrylamide gel (Thermo Fisher Scientific), using a buffer solution NuPAGE MOPS SDS (Thermo Fisher Scientific). The proteins were transferred to a polyvinylidene fluoride(PVDF) membrane through a semi-dry transfer system (Bio-Rad, Hercules, CA, USA). The antibodies used were anti-p65 (Abcam, Cambridge, UK, 1:500), TLR4 (, Santa Cruz Biotechnology, Santa Cruz, CA, USA, 1:500), β-actin ( Sigma-Aldrich, 1:1000) and Histone H3 total (Abcam, 1:20,000). The secondary antibodies were anti-rabbit (p65), anti-mouse (TLR4 and β-actin) and anti-goat (Histone H3 total) (Sigma-Aldrich, 1:1000). The proteins were detected by the chemiluminescent reagent ECL (Western Lightning Chemiluminescence Reagent Plus, Perkin Elmer, Waltham, MA, USA) in the ImageQuant LAS4000 (GE Healthcare).

### Immunofluorescence

Immunofluorescence analysis was performed to analyze p65 and TLR4 distribution in the cell compartments, 0.5 h and 12 h after LPS stimulation, and control with no stimulation. The cells were fixed with methanol and acetone (1:1), the cell membrane was permeabilized with Triton-X-100 (0.1%), and, to avoid unspecific reactions, the cells were treated with 2% bovine serum albumin. The primary antibodies anti-p65 (Abcam, 1:500), anti-TLR4 (Abcam, 1:800) were incubated overnight at 4ºC. The secondary antibodies (Thermo Fisher Scientific) goat anti-Rabbit IgG H&L (Alexa Fluor 488, 1:400) and goat anti-Mouse IgG H&L (Alexa Fluor 546) were incubated overnight, and nuclei were stained with DAPI (Thermo Fisher Scientific, 1:1000 dilution). The preparations were analyzed in confocal microscope Zeiss 780-NLO (Thornwood, NY, USA). The retrieved images were analyzed by Image J/Fiji^58^. The fluorescence values for TLR4 and p65 in the nuclei was delimited by DAPI staining.

### Cell viability analysis

U87MG cells were plated in 96 wells (2 × 10^3^ cell/well), stimulated with LPS alone, Amuvatinib (Amb), a RAD51 inhibitor, alone, TMZ alone and the combined treatment, compared to the non-stimulated, and non-treated group. The plates were observed in four time periods of 24, 48 and 72 h. The concentrations for Amb and TMZ were 10 µM and TMZ 0.54 mM, respectively. The cell viability was analyzed by the Presto Blue reagent (Thermo Fisher Scientific), following the manufacturer instructions. The fluorescence of each well was measured by the Glomax (Promega). The samples were evaluated in octuplicate in two independent experiments, and an additional spot with only the Presto Blue reagent was evaluated to normalize the fluorescence results.

### Apoptosis assay

U87MG cells were treated with LPS and TMZ and the combined treatment LPS and TMZ for 48 h. Cells were washed with PBS and trypsinized. The adherent and non-adherent cells were collected for analysis. Annexin V conjugated with FITC and propidium iodide (PI) were used for the staining following the manufactures instructions (BD Biosciences, San Jose, CA, USA) and the measurements were done by the Flow Cytometry FACs Canto BD (BD Biosciences). The cells presenting the phosphatidylserine translocation with Annexin V positivity were considered as in early apoptosis. The cells with increased membrane permeability resulting in Annexin V and PI positivity were considered as in late apoptosis. The results were analyzed by Flow Jo version 10 (Flow Jo, LLC, Ashton, OR, USA).

### Transcriptome analysis

We performed RNA-Seq to interrogate differentially expressed transcripts induced by LPS treatment for 12 h in the U87MG cell line. Control and LPS-treated cells were analyzed in triplicates. We used the QuantSeq 3′ mRNA-Seq Library Prep kit FWD for Illumina (Lexogen, Vienna, Austria) to generate around 5 million 75 bp single-ended reads per library. Libraries for a given condition (control or LPS) were generated in triplicates. Sequencing was performed in the Illumina NextSeq 550 (Illumina, San Diego, CA, USA) platform at the Sequenciamento em Larga Escala (SELA) facility of FMUSP.

We used STAR to align sequencing data to the GRCh38 version of the human genome (downloaded from ftp.ensembl.org). We used the bamsort tool, from biobambam2, for downstream processing of the BAM file, including merging and sorting. To count the number of reads that overlap each gene, we used featureCounts. We obtained the GFF file containing the gene models from ftp.ensembl.org. We used fastQC and RNASEQC to assess sequencing quality and alignment metrics, respectively. To assess differential gene expression between LPS-treated and control cells, we used the R-Bioconductor package *limma.* Pathway analysis and gene-set enrichment analysis were performed using online tools such as GSEA (http://software.broadinstitute.org/gsea) and WebGestalt (http://www.webgestalt.org/), Gene Ontology Resources (accessed: 2020–02-10 10.5281/zenodo.2529950)^59,60^ and String consortium to produce figure^28^.

### Validation of the transcriptomic analysis

The differentially expressed genes selected from the transcriptomic analysis was validated by qRT-PCRs. The cells were collected in four time points (0.5, 12, 24, 48 h). The genes of the DNA repair pathway analyzed were *UNG, FEN1,* and *RAD51.*

### Gene expression analyses

Gene expression levels were evaluated by qRT-PCR using the Sybr green approach to in an ABI 7500 (Thermo Fisher Scientific), using the Power Sybr green PCR master mix (Thermo Fisher Scientific). The primers were synthesized by IDT (Coralville, IA, USA) and Thermo Fisher Scientific. The sequence of primers were: *TLR4* (F: TTTATCCAGGTGTGAAATCCAGAC; R: TCCAGAAAAGGCTCCCAGG), *TRAF6* (F: TGAAATGTCCAAATGAAGGTTGTT; *R:* GAAGGGACGCTGGCATTG), *MYD88 (*F: GGATGGTGGTGGTTGTCTCTG; R: CCTTGTACTTGATGGGGATCAGT), *SRF* (F: ACAGCAGCACAGACCTCACG; R: CATGCGGGCTAGGGTACATC), *JUN* (F: CTCAGACAGTGCCCGAGATG; R: TTCCTCTCCGCCTTGATCC), *NLRP3* (F: CGGAGACAAGGGGATCAAACT; R: AGCAGCAGTGTGACGTGAGG), *IL1B* (F: GGGACAGGATATGGAGCAACAA, R: TCAACACGCAGGACAGGTACA); *TRIF* (F: AAGCCGTGCCCACCTACTC, R: GAGGAAGGGAACAGGGAGGA); *RIPK1* (F: TTTGGGAAGGTGT CTCTGTGTTT, R: CATCATCTTCGCCTCCTCCA); *RIPK3* (F: CAATATGAATGCTGCTGTCTCCA, R: CCATCCATTTCTGTCCCTCCT), *UNG* (F:TTGCTCTGGGGCTCTTATGC; R:TGACAAAGGGGAGGGATGAG)*, FEN1* (F:GAAGGGAGAGCGAGCTTAGGA; R:GGCAACACAGAGGAGGGATG)*, RAD51* (F:CGTTCAACACAGACCACCAGAC; R:GCGGTGGCACTGTCTACAATAA) *TBP* (F: AGGATAAGAGAGCCACGAACCA; R: CTTGCTGCCAGTCCTGGACTGT), *HPRT* (F: TGAGGATTTGGAAAGGGTGT; R: AGCACACAGAGGGCTACAA), GUSB (F: GAAAATACGTGGTTGGAGAGCTCATT; R: CGAGTGAAGATCCCCTTTTTA). . The expression levels for the tissues samples and cell samples were resulted by the formula 2^−ΔCt^ (where ΔCt = TLR4 Ct—geometric means of three housekeeping genes, TBP, HPRT and GUSB)^27^. The cell samples were analyzed by one housekeeping gene (HPRT).

### In silico analysis using TCGA dataset

Datasets from The Cancer Genome Atlas (TCGA-http://cancergenome.nih.gov/) was downloaded from Genomics Data Commons Data Portal, and the data were normalized by DE-seq. Heatmap z-score of RPKM values were calculated for each gene, and the mean was used as cutoff value to determine whether up- and down-regulated. The TCGA cohorts consist of low grade astrocytomas (63 AGII and 128 AGIII cases), 29 GBM-PN, 38 GBM-CS, and 48 GBM-MES cases.

#### Statistical analysis

The program SPSS version 23.0 (IBM, Armonk, NY, USA) and Graph Pad Prism (GraphPad Software Inc, CA, USA) were used for the statistical analysis and graphs. The Normality of the data distribution was analyzed by the Kolmogorov–Smirnov test. Kruskal–Wallis and post hoc Dunn tests were used to analyze the differences among groups when non-parametric, and One-way Anova when parametric and multiple groups comparisons for the cellular treatment experiments, and Tukey as post hoc test (p65 nuclei staining and expression levels). For multiple variables comparison Two-way Anova was used, upon significance of interaction, column and row factor, Bonferroni post hoc test (apoptosis by flow cytometry) and Tukey post hoc (cellular viability assay) were applied. The test Mann Whitney was used for two groups comparison (TLR4 nuclear staining). The correlations were performed by Spearman's rho test. Differences were considered significant for *p* < 0.05.

## Supplementary information

Supplementary Figures.
