# Supplementary material for: Late p65 nuclear translocation in glioblastoma cells indicates non-canonical TLR4 signaling and activation of DNA repair genes
Source: Sci Rep. 2021 Jan 14;11:1333. doi: 10.1038/s41598-020-79356-1 (PMC7809124; doi:10.1038/s41598-020-79356-1)
Supplement: Supplementary file 1 — Supplementary Figures. [file 41598_2020_79356_MOESM1_ESM.docx]

***Late p65 nuclear translocation in glioblastoma cells indicates non-canonical TLR4 signaling and activation of DNA repair genes***

Isabele F. Moretti^1*^, Antonio M. Lerario^2^, Marina Trombetta-Lima^1^, Paula R. Sola^1^, Roseli da Silva Soares^1^, Sueli M. Oba-Shinjo^1^, and Suely K.N. Marie^1^

1. Laboratory of Molecular and Cellular Biology (LIM15), Department of Neurology, Faculdade de Medicina FMUSP, Universidade de Sao Paulo, SP, BR
2. Department of Internal Medicine, Division of Metabolism, Endocrinology and Diabetes, University of Michigan, Ann Arbor, Michigan, USA.

*Corresponding author e-mail address: imoretti@usp.br

- **Supplementary information**


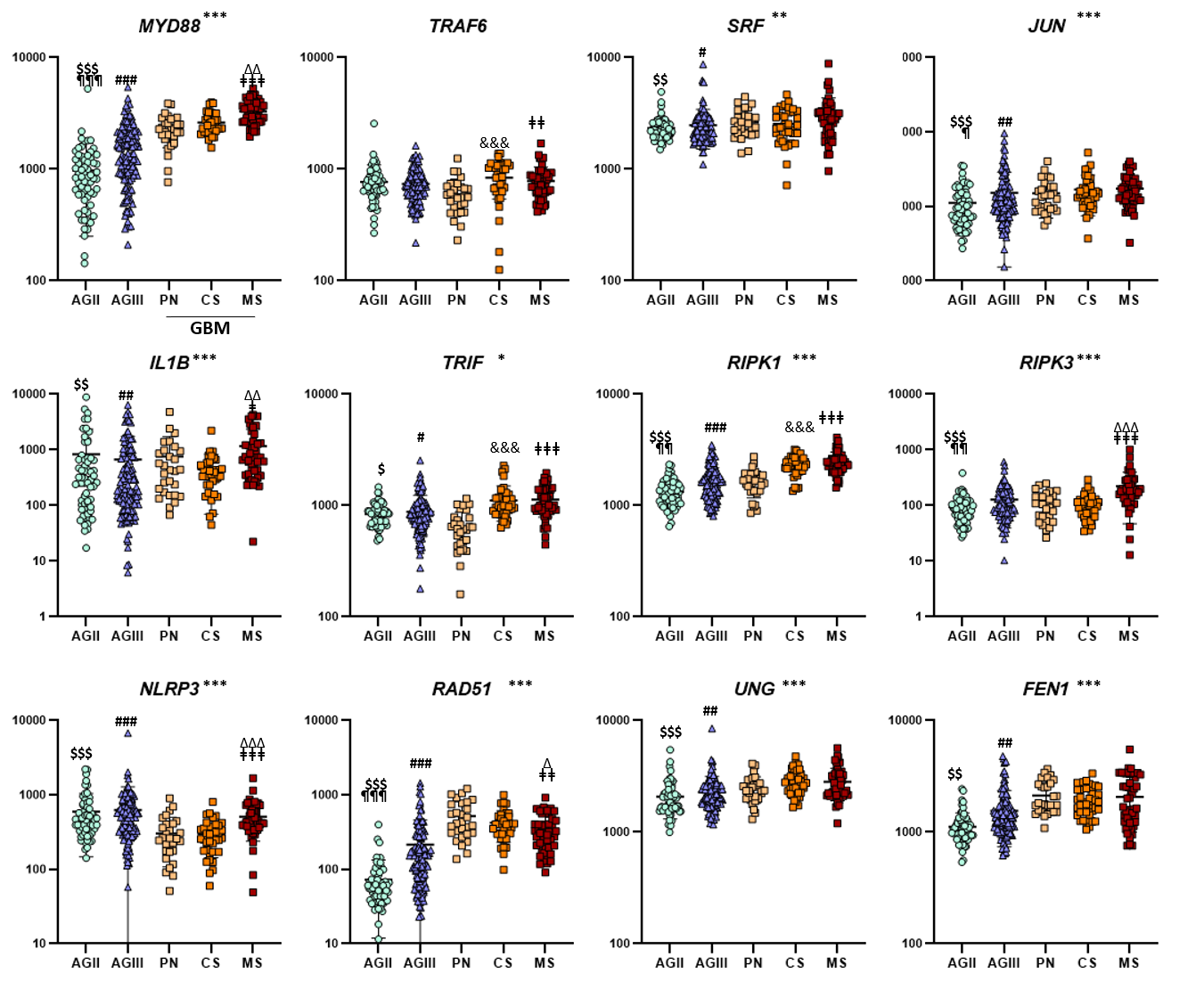


**Supplementary Figure 1.** Expression analysis in the TCGA RNAseq data set for the different genes analyzed throughout the study. For TLR4 canonical and non-canonical pathways targets. The data is demonstrated by reads per kilobase per million mapped reads (RPKM), presented in the graph by log10, including values for low grade astrocytomas and GBM molecular subtypes. Statistical significance were analyzed and demonstrated by (*) for Kruskal-Wallis test, and followed of post hoc Dunn tests, wherein: ¶ AGII in comparison with AGIII; $ AGII in comparison with GBM; # AGIII compared to GBM; (ǂ PN compared to MS; Δ CS compared to MS and & PN compared to CS. The quantity of symbols is proportional to p value, as one symbol p<0.0, two symbols p<0.00, and three symbols p< 0.000.


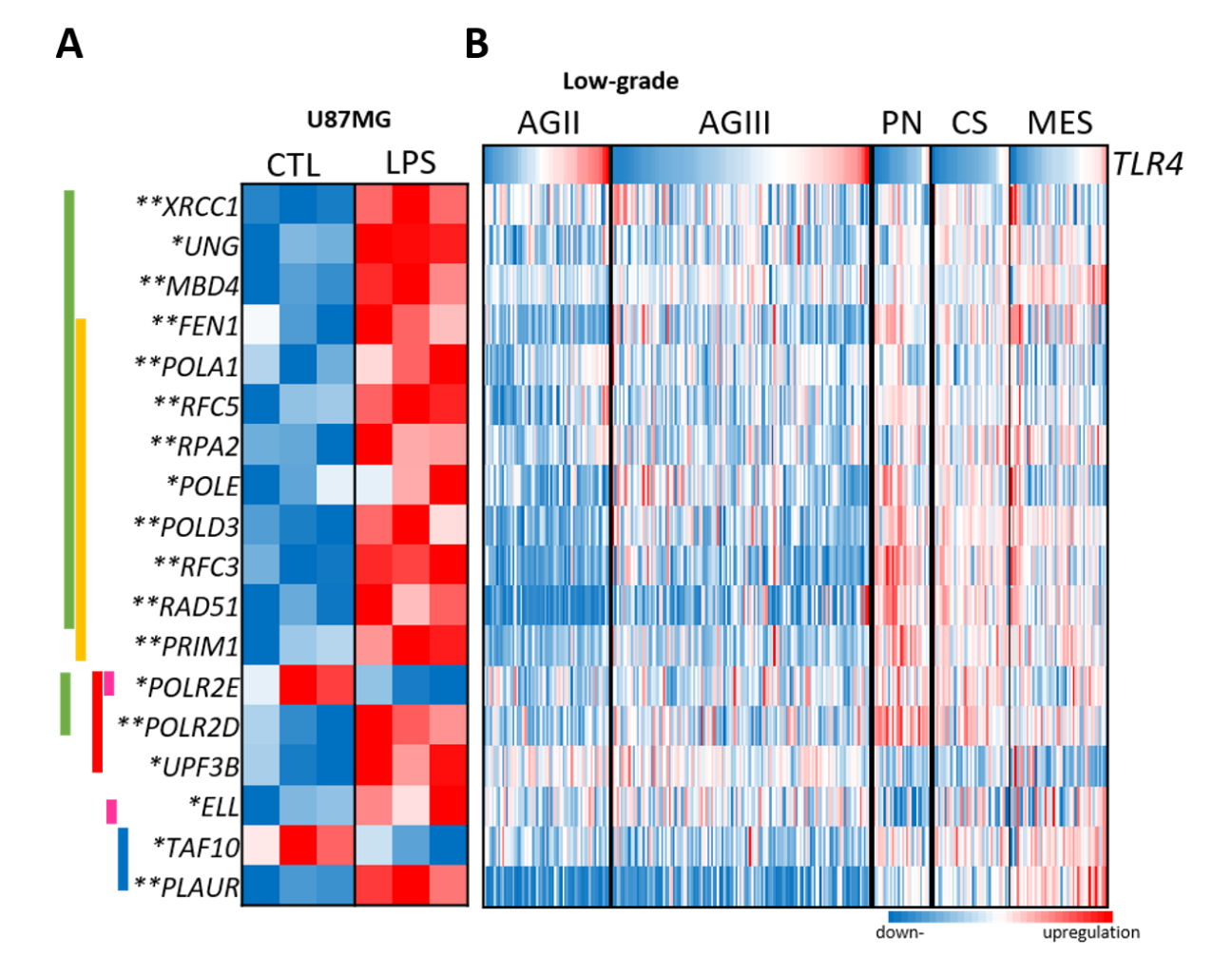


**Supplementary Figure 2**. (A) Heatmap representing the expression of genes coding for proteins with biological functions for DNA repair (genes selected from the gene set enrichment analysis - GSEA). Each group was analyzed in triplicates for CTL and LPS treated. *p-value <0.05 ** adjusted p<0.05 (B) Heatmap representing the expression of selected genes from TCGA RNAseq dataset from astrocytoma cases (AGII, AGIII, and AGIV). GBM cases were subdivided by the molecular subtypes (PN, CS, MES). Up-regulated values are in red and down-regulated in blue. The log2CPM values were normalized by z-score.


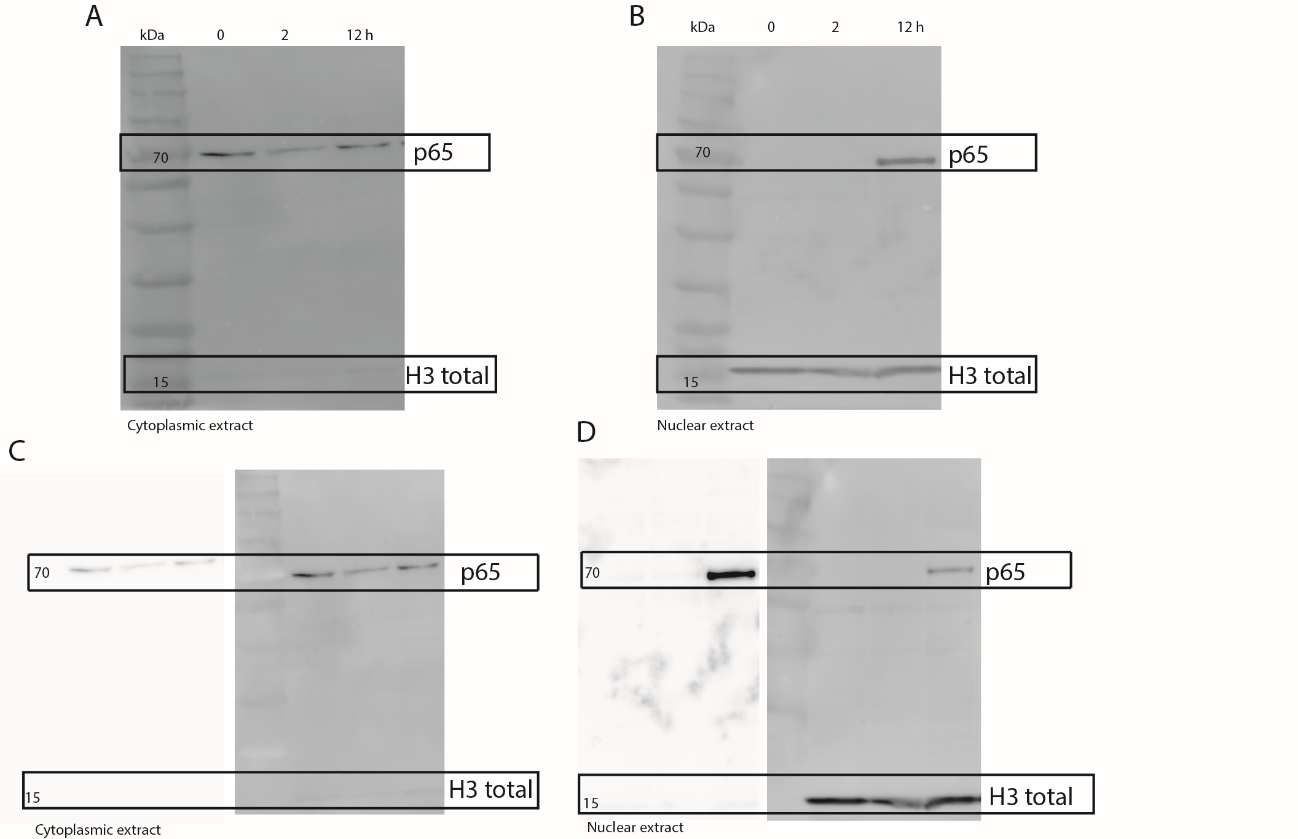


**Supplementary Figure 3**- Western blot full length images highlighting the areas presented in figure 3 B. Both images were merged with the protein ladder for size localization. (A) Cytoplasmatic extract incubated with anti-p65 and anti -H3 total and (B) nuclear extract incubated with anti-p65 and anti -H3 total. The antibodies were incubated in different times in the same membrane in (C) Cytoplasmatic extract for anti-p65, left membrane exposed for 1 minutes and anti-H3 total right membrane, exposed for 1 minute and (D) nuclear extract for anti-p65, left membrane, exposed for 2 minutes and anti-H3 total right membrane, exposed for 1 minute.


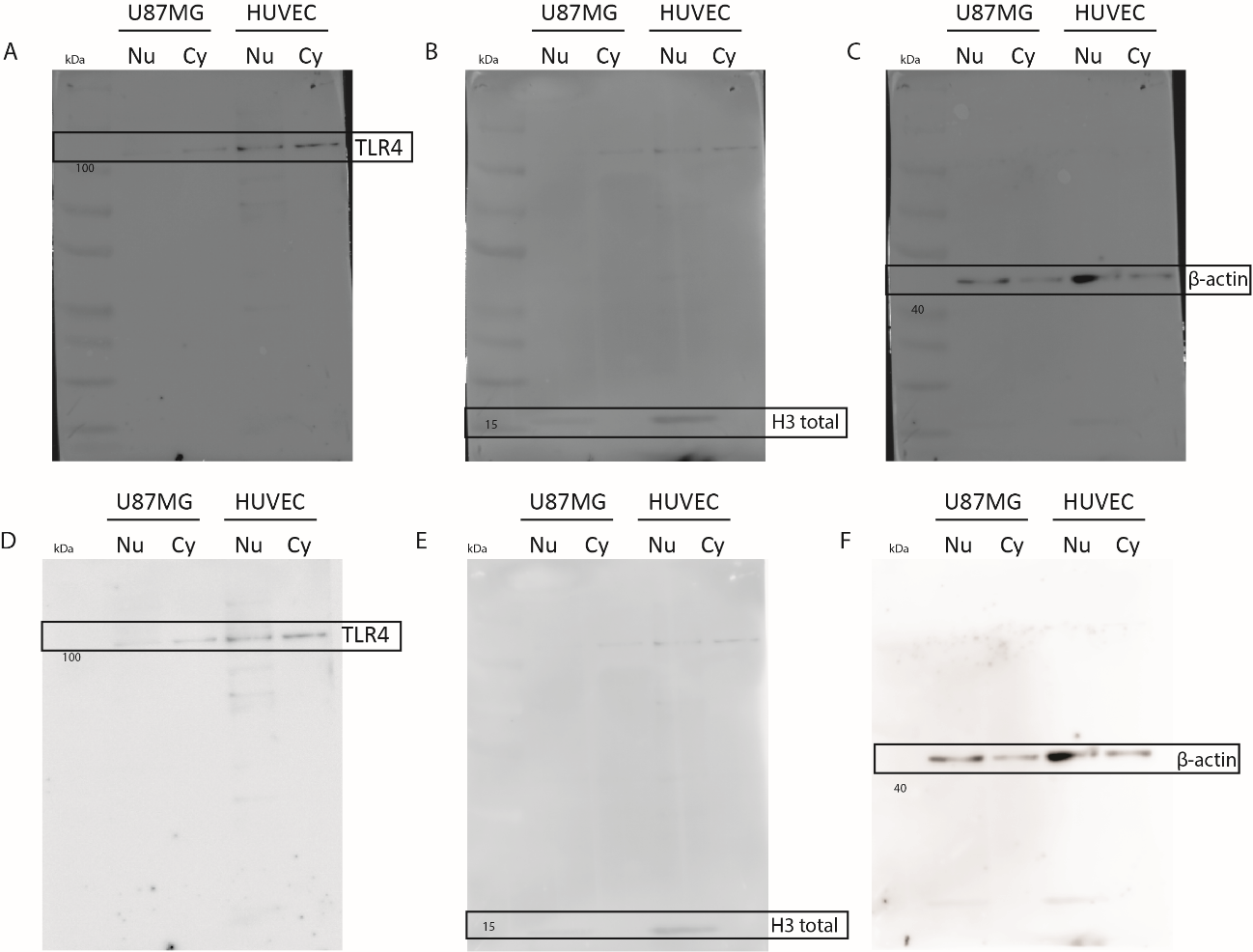


**Supplementary Figure 4-** Western blot full length images highlighting the areas presented in figure 6 D merged with the protein ladder image, the antibodies were incubated in different times in the same membrane in (A) anti-TLR4 first incubated antibody with the membrane exposition for 5 min. In (B) anti-H3 total image exposed for 1min30s and (C) anti-β-actin exposed for 1 minute. (D-F) Same images without the ladder image merged.
